# Pathway Analysis and Metabolites Identification by Metabolomics of Etiolation Substrate from Fresh-Cut Chinese Water Chestnut (*Eleocharis tuberosa*)

**DOI:** 10.3390/molecules21121648

**Published:** 2016-12-01

**Authors:** Yi-Xiao Li, Yong-Gui Pan, Feng-Ping He, Meng-Qi Yuan, Shang-Bin Li

**Affiliations:** College of Food, Hainan University, Haikou 570228, China; lisa19910@163.com (Y.-X.L.); hfping123@126.com (F.-P.H.); sullna@163.com (M.-Q.Y.); sharplee1208@163.com (S.-B.L.)

**Keywords:** Chinese water-chestnut, metabolomics, LC–MS, metabolites, pathway analysis

## Abstract

Fresh-cut Chinese water chestnuts (CWC) turn yellow after being peeled, reducing their shelf life and commercial value. Metabolomics, the systematic study of the full complement of small molecular metabolites, was useful for clarifying the mechanism of fresh-cut CWC etiolation and developing methods to inhibit yellowing. In this study, metabolic alterations associated with etiolation at different growth stages (0 day, 2 days, 3 days, 4 days, 5 days) from fresh-cut CWC were investigated using LC–MS and analyzed by pattern recognition methods (principal component analysis (PCA), partial least squares-discriminant analysis (PLS-DA), and orthogonal projection to latent structures-discriminant analysis (OPLS-DA)). The metabolic pathways of the etiolation molecules were elucidated. The main metabolic pathway appears to be the conversion of phenylalanine to *p*-coumaroyl-CoA, followed by conversion to naringenin chalcone, to naringenin, and naringenin then following different pathways. Firstly, it can transform into apigenin and its derivatives; secondly, it can produce eriodictyol and its derivatives; and thirdly it can produce dihydrokaempferol, quercetin, and myricetin. The eriodictyol can be further transformed to luteolin, cyanidin, dihydroquercetin, dihydrotricetin, and others. This is the first reported use of metabolomics to study the metabolic pathways of the etiolation of fresh-cut CWC.

## 1. Introduction

Metabolomics, a new field in systems biology, represents an emerging and powerful discipline concerned with the comprehensive analysis of small molecules (<1 kDa); it provides a powerful approach for the discovery of biomarkers in biological systems [1,2,3,4]. Metabolomics provides a quantitative analysis of all low-molecular-mass metabolites and lends insight into the relationship between metabolites and changes in physiology/pathophysiology status in biochemical networks and pathways by combining a range of different analytical technologies and calculation methods [5,6,7]. These techniques have recently demonstrated significant potential in many fields, such as toxicology [8], nutrition [9], environmental stress [10], global effects of genetic manipulation, disease diagnosis, drug mechanism and development, natural product discovery, and comparison of different growth stages [11,12]. Additionally, metabolomics has been used in food science to assess food quality, food safety, food microbiology, and food processing [13,14,15]. Furthermore, the global metabolite profile now enables much more detailed studies of noninvasive biomarkers that can detect different growth stages in plants.

Plant studies using metabolomics techniques aim for the simultaneous detection of all metabolites in plant tissues. Metabolomics detection technology includes nuclear magnetic resonance (NMR) spectroscopy, liquid chromatography-mass spectrometry (LC–MS) [16], and gas chromatography–mass spectrometry (GC–MS). Among these, GC–MS is mainly suited for compound classes appearing mainly in primary metabolic pathways, such as amino acids, fatty acids, carbohydrates, and organic acids [17]. LC–MS is more suitable for assessing the overall biochemical richness of plants. This technique analytically covers the large (semipolar) group of plant secondary metabolites including alkaloids, saponins, phenolic acids, phenylpropanoids, flavonoids, glucosinolates, polyamines, and their derivatives [18,19]. Therefore, LC–MS is preferred over the other techniques in the study of numerous structure databases [20]. At present, LC–MS has become one of the frequently used techniques in metabolomics studies because of its high sensitivity and reproducibility.

The Chinese water chestnut (CWC, *Eleocharis tuberosa*) is one of the most popular hydrophytic vegetables in China because of its unique taste [21]. However, it is usually peeled before being eaten, and fresh-cut CWC turns yellow after being peeled, reducing its shelf life and its commercial value. Recent studies have shown that CWC etiolation is linked to the presence of eriodictyol and naringenin [21]. In addition, most researches have shown that phenylalanine ammonia-lyase (PAL) was present in fresh-cut CWC tissue. With the extension of storage time, the activity of PAL increases and the etiolation of CWC also increases. Therefore, the metabolic pathways of eriodictyol and naringenin are believed to be associated with PAL activity [22]. PAL is a critical and rate-limiting enzyme in the phenylpropanoid metabolic pathway, a pathway that also contains eriodictyol and naringenin [23,24,25,26]. It is therefore probable that the etiolation process of fresh-cut CWC is related to the functioning of the phenylpropanoid metabolic pathway. However, there may be a variety of specific metabolic pathways related to yellowing, and few prior studies have investigated these pathways fully.

The synthesis of flavonoids begins with the shikimic acid metabolic pathway, which generates phenylalanine. This molecule then enters the phenylpropanoid metabolic pathway, and then, through the common enzyme chalcone synthase, enters the flavonoid synthesis pathway, which generates the various flavonoids through different branches [27,28]. Though the use of metabolomics to research flavonoids is relatively uncommon, both naringenin and eriodictyol are considered flavonoids, and the molecular weight of most flavonoids is below 1 kDa; therefore, the metabolites of the etiolation process are suitable for studying with metabolomics techniques. Literature about the process of involving metabolomics in the research of flavonoids is very common, especially in herbal aspects [29,30]. In terms of fruit and vegetables, the glycosides of luteolin and apigenin as well as quercetin conjugates were identified from *Phoenix dactylifera* (date palm) fruits using UPLC/PDA/ESI-TOF-MS methods [31,32]. Flavonoids in blueberry extracts were analyzed through HPLC-ESI-MS/MS, which identified that anthocyanins and flavonoid alcohols are the main components [32]. Therefore, there is precedence for identifying the characteristic flavonoids and metabolic pathways from plants.

In order to clarify the mechanism of fresh-cut CWC etiolation and yellowing, as well as to evaluate the safety of the etiolation metabolite, we sought to discover the metabolic pathways of the molecules responsible for etiolation. In this study, we used the LC–MS method combined with additional analytical technologies and calculation methods (principal component analysis (PCA), partial least squares-discriminant analysis (PLS-DA), orthogonal projection to latent structures-discriminant analysis (OPLS-DA)) to analyze the metabolic pathways of etiolation in fresh-cut CWC.

## 2. Results and Discussion 

### 2.1. LC–MS Analysis of Metabolic Patterns

In this study, five time points from the yellowing process of CWC were studied using an LC–MS metabolomics approach. For the analysis, we compared 0 day (see Methods) with 2 days (0–2), 2 days with 3 days (2–3), 3 days with 4 days (3–4), and 4 days with 5 days (4–5) for screening and identification of the metabolites. According to peak height data, we detected 1046 positive-ion (ESI+) peaks and 1962 negative-ion (ESI−) peaks by Mass Profiler using the same acquiring method of 0.5–15 min of retention time, and then the normalized data were fed to SIMCA-P. The parameters (R^2^X, R^2^Y, and Q^2^Y) obtained from SIMCA-P indicated that the model was able to distinguish between each of the yellowing time points in both ion modes.

**Figure 1:**
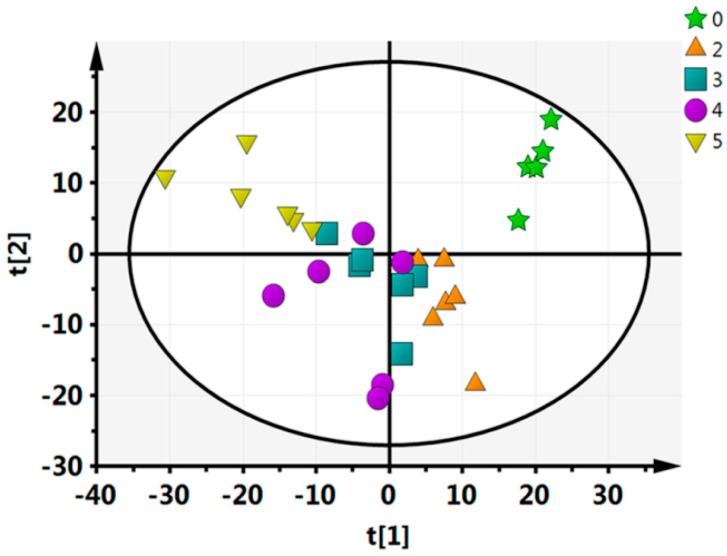
Statistical principal component analysis (PCA) analysis of the 5 time points.

**Figure 2:**
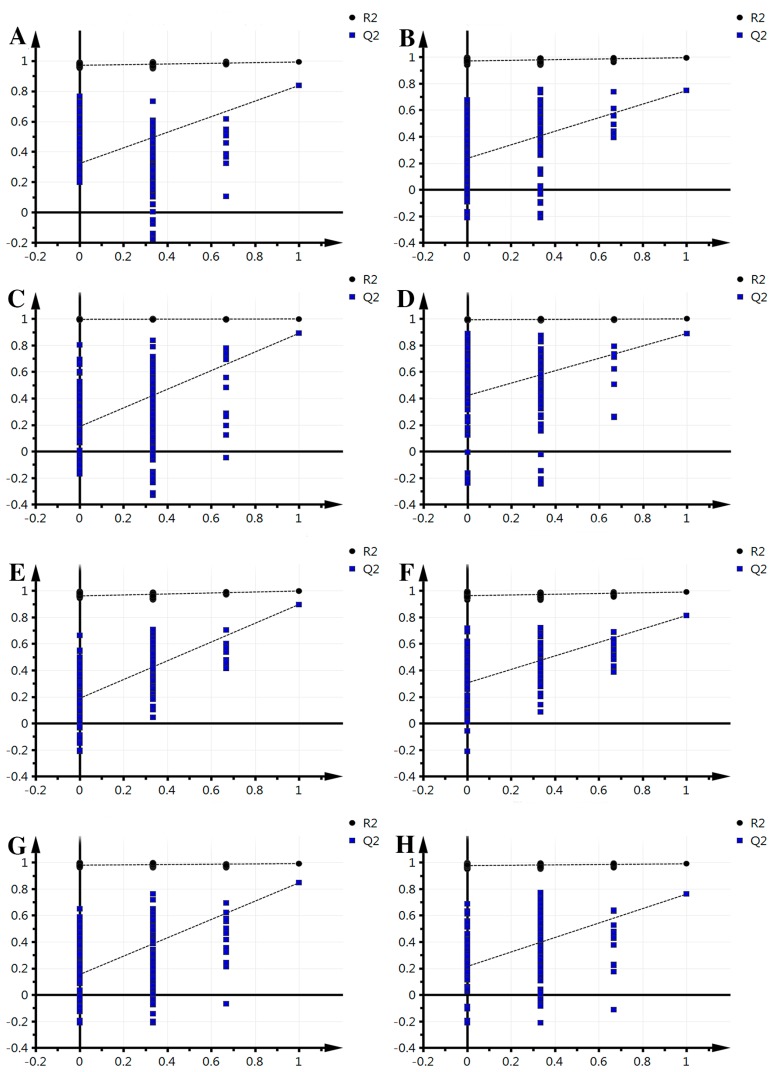
Effective verification of fresh-cut Chinese water chestnut during different storage times. (**A**) 0–2 comparison, ESI+; (**B**) 2–3 comparison, ESI+; (**C**) 3–4 comparison, ESI+; (**D**) 4–5 comparison, ESI+; (**E**) 0–2 comparison ESI−; (**F**) 2–3 comparison, ESI−; (**G**) 3–4 comparison, ESI−; (**H**) 4–5 comparison, ESI−.

To further evaluate the capability of the LC–MS-based metabolomics approach to distinguish between all of the time points, multivariate data analyses (PCA, PLS-DA, OPLS-DA) were performed. In the PCA analysis, we identified five principal components in positive mode (R^2^X = 0.464, Q^2^ = 0.124) (Figure 1). For this nonsupervisory PCA model, the R^2^X parameter-which describes the rate of the model-can be used to determine the model’s quality. Generally, a model is reliable when R^2^X > 0.4 [33]. Thus, the current PCA model was suited to explain the metabolic differences between each group. Every point in Figure 2 represents a sample with the fresh-cut CWC; from the PCA analysis it can be visually seen that there is a significant difference between each pair of time points, except for between 3 days and 4 days. Furthermore, the between-group differences were significantly greater than the intergroup differences, indicating that substantial biochemical perturbation occurred in all groups. PCA as an unsupervised analysis method, responsive to the primitive state of the data, can be used to observe the relationship between each group, but it cannot account for intergroup errors or eliminate the random error unrelated to this research. In order to obtain information on which metabolites were the cause of the differences, we conducted PLS-DA and OPLS-DA analyses for each pair of time points.

The supervised PLS-DA analysis revealed greater differences in the metabolic signature between each pair of time points, focusing on intergroup differences and minimizing between-group differences to better grasp the overall characteristics and variation of the data. For each pair of time points studied, this analysis was performed in both the positive mode and negative mode. Results for each pair are as follows (Figure 2): in positive mode, groups 0–2 obtained one principal component (R^2^X = 0.253, R^2^Y = 0.97, Q^2^ = 0.845); groups 2–3, one principal component was obtained (R^2^X = 0.328, R^2^Y = 0.923, Q^2^ = 0.68); groups 3–4 obtained one principal component (R^2^X = 0.179, R^2^Y = 0.897, Q^2^ = 0.588); groups 4–5, one principal component was obtained (R^2^X = 0.198, R^2^Y = 0.867, Q^2^ = 0.514); in negative mode, groups 0–2 obtained one principal component (R^2^X = 0.278, R^2^Y = 0.945, Q^2^ = 0.829); 2–3, one principal component was obtained (R^2^X = 0.298, R^2^Y = 0.913, Q^2^ = 0.787); groups 3–4 obtained two principal components (R^2^X = 0.321, R^2^Y = 0.991, Q^2^ = 0.806); groups 4–5, one principal component was obtained (R^2^X = 0.259, R^2^Y = 0.879, Q^2^ = 0.712). The model parameters (R^2^X and Q^2^) indicate that the current PLS-DA models were reliable and suitable for interpretation between each pair of time points of metabolic differences and finding the differences. Meanwhile, the left of the arrange experiments were randomly generated depending on whether the value of R^2^ and Q^2^ was less than the original value from the right. This indicates that the predictive power of the original model is greater than any randomized *y* variables, thus, the model is effective and it can continue to be applied to the different metabolites.

**Figure 3:**
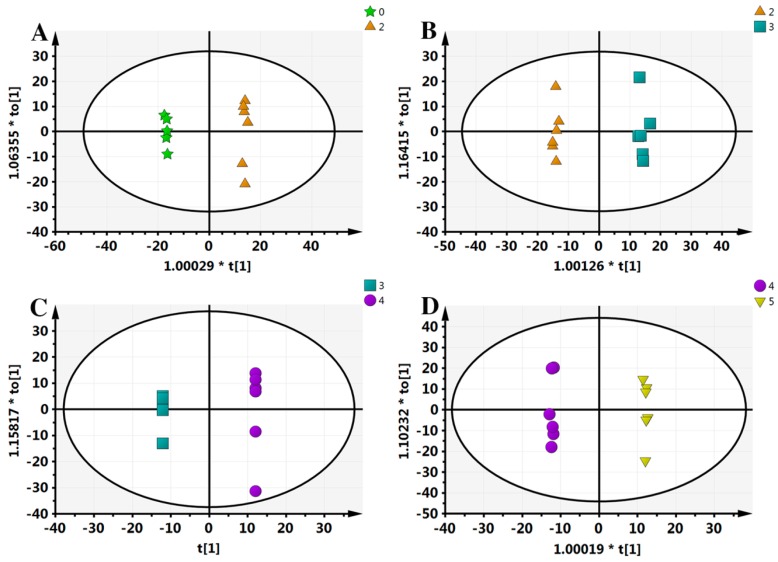
Orthogonal projection to latent structures-discriminant analysis (OPLS-DA) results of all samples. (**A**) 0–2 comparison, scanned by ESI+; (**B**) 2–3 comparison, ESI+; (**C**) 3–4 comparison, ESI+; (**D**) 4–5 comparison, ESI+. “*” means the sample separation significantly.

Typically, OPLS-DA analysis is conducted to search for the different components that lead to the maximum differences between two samples. In order to further obtain the metabolic differences, this study applied an OPLS-DA approach; separating samples into two blocks. As can be seen from Figure 3, compared with PCA analysis, each group received the highest degree of separation, which is useful to determine the metabolic differences.

### 2.2. Identification of Metabolites

All metabolites from each total ion chromatogram were extracted and aligned using Mass Profiler software. All information, including retention time, peak intensity, and exact mass, was supplied by the LC-Q/TOF-MS platform. The metabolites (VIP (variable importance in the projection) > 1) were analyzed by *t*-test, and metabolites with a *p*-value < 0.05 were identified as metabolic differences.

#### 2.2.1. Identification of Metabolites in 0–2 Days

Comparing the samples of 0 day with 2 days, the main metabolite differences are shown in Table 1 and Table 2. In the positive mode, 5′-deoxy-5′-(methylthio) adenosine, ferulic acid, and linolenic acid showed significant increases, while *N*-palmitoyl threonine and stearidonic acid decreased. Among these, the linolenic acid at 2 days compared with 0 day increased by about 19 times. In the negative mode, salicylaldehyde, salicylic acid, naringenin, eriodictyol, pentahydroxyflavanone, apigenin-glucuronide, and luteolin 5-methyl ether 7-glucoside were increased significantly. The naringenin has increased by about 4820 times and the eriodictyol was increased by about 700 times, while the PE (phosphatidyl ethanolamine) (18:3), PE (18:1), and quercetin 3-*O*-(6″-malonyl-glucoside) 7-*O*-glucoside at 2 days compared with 0 day were decreased by 600 times. Thus, the metabolites between 0 day and 2 days have significant differences, and these metabolites were related to the pathways of fresh-cut CWC yellowing.

#### 2.2.2. Identification of Metabolites in 2–3 Days

Comparing the samples of 2 days with 3 days, the main metabolite differences are shown in Table 3 and Table 4. In the positive mode, cyanidin 3-*O*-(6-*O*-malonyl-β-d-glucoside) and dihydroxy-6,7,4′,5′-tetramethoxyflavone increased significantly, while aconitic acid and MG (18:2) decreased. In the negative mode, phenylacetic acid, vanillic acid, quinic acid, quercetin and its derivatives, dihydroxy-dimethoxy-methylenedioxy, the derivatives of naringenin, malvidin, and luteolin were increased significantly, all of them showing an average increase of about 280 times. In contrast, ascorbic acid and arginine at 3 days, compared with 2 days, were decreased by 4 times. The major differences were identified to further explore the pathway of CWC yellowing.

#### 2.2.3. Identification of Metabolites in 3–4 Days

Comparing the samples of 3 days with 4 days, the main metabolite differences are shown in Table 5 and Table 6. Overall, the metabolic differences between 3 days and 4 days were few. In the positive mode, dihydroxy-7,2′-dimethoxyflavanone at 4 days compared with 3 days was increased by about 3.5 times. On the other hand, trihydroxy-3-methoxyflavone decreased by about 12 times. From the negative mode, kolaflavanone, eriodictyol, the derivative of naringenin, cyanidin, and myricetin were increased. In contrast, linolenic acid and phenylacetic acid at 4 days, compared with 3 days were decreased.

#### 2.2.4. Identification of Metabolites in 4–5 Days

Comparing the samples of 4 days with 5 days, the main metabolite differences are shown in Table 7 and Table 8. In the positive mode, dehydrophytosphingosine at 5 days, compared with 4 days, increased by about 5400 times. From the negative mode, naringenin-4’-*O*-β-d-glucuronide changes significantly. At 5 days, compared with the 4 days, it fell by about 110 times; other metabolic changes were small.

### 2.3. Biological Pathway and Function Analysis

**Figure 4:**
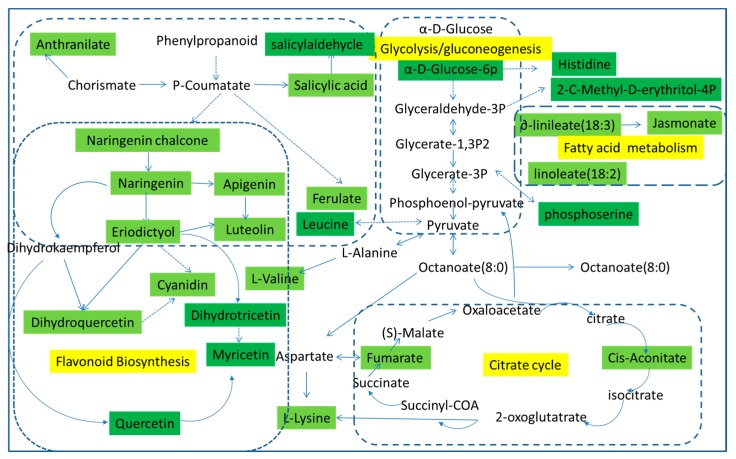
Metabolomic Pathway Analysis (MetPA) construction of the metabolic pathways involved in the etiolation of fresh-cut CWC.

Using pattern recognition analysis on the metabolites, we were able to achieve a clear separation between each pair of time points. To identify the pathways involved in fresh-cut CWC etiolation, previously identified metabolites were analyzed using MetPA (Metabolomic Pathway Analysis) software. MetPA is a web-based tool that derives much of its power from the KEGG metabolic pathways database. In metabolic networks, changes in more “important” positions result in a more substantial effects on the pathway compared to changes occurring in marginal or relatively isolated positions. In our analysis, we identified several pathways that appear to be involved in etiolation, including the glycolysis pathway, fatty acid metabolic pathway, citrate cycle pathway, and flavonoid biosynthesis pathway. The green parts in Figure 4 represent the differences we have detected. In the glycolysis pathway, the α-d-glucose is converted to α-d-glucose-6p and then further transformed into glyceraldehyde-3P, while the α-d-glucose-6p is converted to histidine; glyceraldehyde-3P and glycerate-1,3P2 are convert to each other, and at the same time converted to 2-*C*-methyl-d-4-erythritol-4P. Glycerate-3P and phosphoenol-pyruvate are convert to each other and generate phosphoserine. Pyruvate is created from phosphoenol-pyruvate, and converts to octanoate (8:0), triggered the citrate cycle pathway. Furthermore, pyruvic, leucine, and l-alanine interactions in the flavonoid biosynthesis pathway have a certain effect.

**Figure 5:**
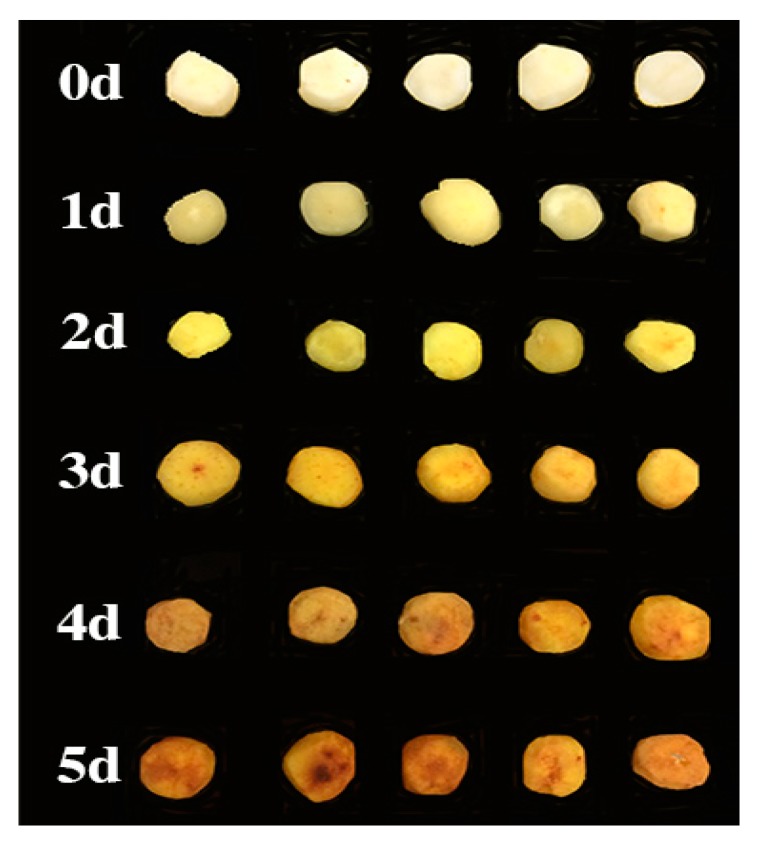
The yellowing process from the fresh-cut Chinese water chestnut. Each day has 5 replicate samples.

The major substance that causes the fresh-cut CWC yellowing are naringenin and eriodictyol, both found in the flavonoid biosynthesis pathway. Therefore, the flavonoid biosynthesis pathway is the most important pathway for CWC yellowing. As we can see from Figure 5, phenylalanine is converted to p-coumaroyl-CoA, followed by conversion to naringenin chalcone, to naringenin, and then naringenin enters different pathways. Firstly, it can transform into apigenin and its derivatives; secondly, it can produce eriodictyol and its derivatives; and thirdly it can produce dihydrokaempferol, quercetin, and myricetin. From here, eriodictyol can be further transformed to luteolin, cyanidin, dihydroquercetin, dihydrotricetin, and others. According to the metabolism pathway, quercetin, myricetin, apigenin, luteolin, and other substances were also yellowing substances. Therefore, the yellowing of fresh-cut CWC is caused by several yellow substances, and our study indicated that the flavonoid biosynthetic pathway is the main mechanism for fresh-cut CWC yellowing. Detailed construction of the yellowing metabolism pathway is shown in Figure 5.

## 3. Materials and Methods

### 3.1. Chemicals, Reagents and Materials

Acetonitrile (LC/MS grade) and methanol (HPLC grade) were purchased from Merck (Damstadt, Gemany). Formic acid was purchased from CNW (Düsseldorf, Gemany). Leucine-enkephalin was obtained from Sigma Company (San Francisco, CA, USA). Distilled water was purchased from Watsons (Hong Kong, China). NaClO were purchased from “Xiya” Company (Chengdu, China).

Fresh CWCs were purchased in Haikou, China and stored in the laboratory at 17 °C. Fruits without physical damage or diseases and with uniform size were selected for analysis. CWCs were treated as reported by You [34] and Peng [35]. Fresh CWCs (100 kg) were washed, peeled using a sharp, stainless steel knife, and chopped into small, thick slices. The slices were then disinfected with 0.1% NaClO for 15 min before airing. The CWCs were packed with plastic packaging machines and preserved at 17 °C for 5 days.

To assess the primary metabolites as the CWCs turn yellow, CWCs were randomly selected from five different yellowing stages (Figure 5): one hour post-cutting (0 day), two days post-cutting (2 days), three days post-cutting (3 days), four days post-cutting (4 days), and five days post-cutting (5 days). Slices taken from the yellowed surface were then used as samples. Since the sample between 0 day and 1 d showed little difference, the sample of 1 d was removed from further analyses. Six samples from each of the five time stages (0.5 g each) were transferred to a 1.5 mL centrifuge tube, mixed with 1 mL solvent (MeOH/H_2_O, 7:3 *v*/*v*) by smashing for 1 min, extracted by ultrasonication for 10 min, and then separated by centrifugation at 12,000 rpm at 4 °C for 15 min. Finally, 100 μL aliquots of the supernatant were transferred to autosampler vials.

### 3.2. Metabolic Profiling and Data Analysis

Chromatographic analysis was performed on an Agilent C18 column (Agilent, 100 mm × 2.1 mm, 1.8 μm) using an Agilent LC system (Agilent, 1290 Infinity LC, 6530 UHD, Palo Alto, CA, USA). The column temperature was maintained at 40 °C and eluted at a flow rate of 0.4 mL/min. The mobile phase consisted of a linear gradient system of (A) 0.1% formic acid in water and (B) 0.1% formic acid in acetonitrile. The gradient elution process: 0–3 min, 20%–50% B; 3–13 min, 50%–95% B; 13–15 min, 95% B. The injection volume was 4 μL and the autosampler temperature was 4 °C.

The eluent was introduced into the mass spectrometer (Agilent 6530 Accurate-Mass Q-TOF/MS). The MS system was operated using positive-ion (ESI+) and negative-ion (ESI−) modes. Conditions of positive-ion analysis were as follows: nitrogen was used as the nebulizing gas; the cone gas flow was set at 50 L/h, and the desolvation gas flow was set at 600 L/h. The capillary voltage was 4.0 kV, the sampling cone voltage was 35.0 kV, the source temperature was 100 °C, the desolvation temperature was 350 °C, and the extraction cone voltage was 4.0 V. In negative-ion mode, the cone gas flow was set at 50 L/h and the desolvation gas flow was set at 700 L/h. The capillary voltage was 3.5 kV, the sampling cone voltage was 50 kV, the source temperature was 100 °C, the desolvation temperature was 300 °C, and the extraction cone voltage was 4.0 V. The scan time was set to 0.03 s with a 0.02 s interscan time. Data were collected from 50 to 1000 *m*/*z*. For accurate and repeatable mass acquisition, a lock-mass of leucine-enkephalin was used via a lock spray interface monitor for positive-ion mode ([M + H]^+^ = 556.2771 Da) and negative-ion mode ([M − H]^−^ = 554.2615 Da) to ensure accuracy during the MS analysis.

All data were processed using Mass Profiler in the MassHunter software platform (Agilent) for LC/MS data preprocessing, followed by manual postediting in Microsoft Excel. We removed impurity peaks due to column bleeding and sample preparation. The intensity of each ion was normalized with respect to the total ion count to generate a data matrix that consisted of the rt_mass (retention time_mass), the quantity of observe, and the normalized peak. After that, the multivariate data matrix was introduced into SIMCA-P 13.0 software (Umetrics, Umeå, Sweden) for PCA. PCA data were visualized by plotting the PC scores, with each point representing one rt_mass pair. Thus, the loading plot gives an indication of the metabolites that most strongly influence the patterns in the score plot. In order to further discriminate the metabolites and biomarkers, the data were analyzed using PLS-DA and OPLS-DA methods. Variables that significantly discriminated between groups were considered potential biomarkers and were assigned with *p*-values using a student’s test (*t*-test); *p*-values less than 0.05 were considered statistically significant [36]. The identities of specific metabolites were confirmed by comparison of their mass spectra and mass-to-charge ratio (*m*/*z*) with free online databases, including METLIN (http://metlin.scripps.edu/).

The construction, interaction, and pathway analyses of potential biomarkers were performed with MetaboAnalyst 2.0, with database sources, including METLIN and KEGG (http://www.genome.jp/kegg/), to help identify pathways that were most significantly altered.

## 4. Discussion

The yellowing of fresh-cut CWC is one of the leading causes of reduced shelf life and commercial value, and it is known to be enzymatically different from the browning of other vegetables and fruits [37,38,39]. Despite attempts to reduce CWC yellowing [37,40], we still lack methods to fundamentally inhibit etiolation, and the mechanism of yellowing remains unclear. The advent of the metabolomics field represents a paradigm shift in metabolic research, away from methods that focus on a limited number of single pathways and towards approaches that capture the entire complexity of metabolic networks [41]. Emerging techniques in metabolomics provide a powerful platform for discovering novel biomarkers and biochemical pathways [42]. This may allow us to develop techniques that inhibit CWC yellowing, potentially leading to future advances in inhibition of these pathways.

In this study, samples from different time points of CWC yellowing were analyzed by LC-Q/TOF-MS, coupled with multivariate data analyses; the major metabolic differences were obtained. All samples were successfully modeled, and we identified significant differences in PCA scores between each pair of time points. The pattern was verified as authentic by the PLS-DA analysis. Then, the difference between each pair of time points was analyzed by OPLS-DA combined with *t*-test, and the metabolites were obtained. It was found that metabolic differences include organic acids (such as salicylic acid, citric acid, ferulic acid, leucine, l-aspartic acid, lysine etc.) and flavonoids (like eriodictyol, naringenin, quercetin, myricetin, apigenin, luteolin etc.), while sucrose mainly existed in negative mode. Among those mentioned, two of the flavonoids were yellowing substances. The content changes of eriodictyol and naringenin is outstanding, which showed an upward trend that was proportional to the degree of fresh-cut CWC yellowing during the storage time. The change of myricetin showed a moderate upward trend during storage. In comparison with eriodictyol and naringenin, the change in apigenin and luteolin showed less of an upward trend and did not rise after that time, and the content of quercetin showed fluctuations in growth, which is currently under further investigation. It was demonstrated that the yellowing phenomenon of fresh-cut CWC was caused by a series of yellowing substances, mainly eriodictyol and naringenin, which is consistent with the study of Pan [21]; the content of flavonoids that cause yellowing was proportional to the degree of fresh-cut CWC yellowing. Nowadays, flavonoids are known to be key antioxidant substances that have antiaging, disease resistance, antimicrobial, anti-inflammatory, and antitumor activities, and are becoming more and more abundant in modern life. This study aims to lay the foundation for the further study of fresh-cut CWC yellowing by investigating the substances causing the phenomenon [43]. It was proved that yellowing substances of fresh-cut CWC were connected with the glycolysis pathway, fatty acid metabolism pathway, citrate cycle pathway, and flavonoid biosynthetic pathway by identifying metabolic differences combined with METLIN and KEGG data. Among the metabolites identified, organic acids began to accumulate in the initial stage of the fresh-cut CWC yellowing and were consumed as respiratory matrixes of the glycolysis pathway and citrate cycle pathway in their last stages. Organic acids also participate in respiration and metabolic processes of phenols, amino acids, esters, and flavonoids [44], explaining that organic acids increased gradually at the early time points and descend gradually at the end.

The flavonoids causing the yellowing of fresh-cut CWC mainly derived from the flavonoid biosynthetic pathway. It was proved that phenylalanine was converted into *p*-coumaric-CoA in the first step, which consistent with the study of Burbulis [45]. Naringenin, which is a significant checkpoint in the flavonoid biosynthetic pathway, is converted into the second key substance, eriodictyol, which was converted into dihydrokaempferol, then quercetin, and finally, myricetin. In the metabolic pathway, luteolin, apigenin and its derivatives, quercetin, and myricetin were also yellowing substances, in addition to naringenin and eriodictyol, which revealed that the yellowing is the result of the joint action of flavonoids, mainly naringenin and eriodictyol. This suggests that the etiolation molecules are a mixture of metabolites, but it is clear that the main substances are naringenin and eriodictyol. According to other research reports, in the tissue of grape and corn, 4-coumarin-CoA is generated by phenylalanine in the phenylpropanoid metabolic pathway, which is converted into naringenin chalcone, then naringenin, eriodictyol, and pentahydroxyflavanone were generated in the end [46]; these reports are consistent with this study. The pathway of fresh-cut CWC yellowing is complex, and each pathway contains multiple metabolites; there are rare reports about the relationship between the intermediate products and their interaction mechanism that leads to yellow that it is a complex pathway. In the further work, an in vitro model will be set up to simulate the process of flesh-cut CWC yellowing to confirm the reliability of the metabolic pathways.

## 5. Conclusions

To the best of our knowledge, this is the first report of a metabolomics study of the etiolation of fresh-cut CWC. A rapid, reliable, and sensitive LC-Q/TOF-MS method was validated for qualitative analysis of the different stages of CWC yellowing. Metabolites from each time point could be distinguished from each other by this method, which demonstrates that a significant metabolic perturbation is occurring. These 3008 peaks (1046 peaks in positive mode and 1962 in negative mode) could be useful as biomarkers for the different stages of yellowing. Interestingly, the flavanoid metabolic pathway emerged as a key pathway in the etiolation of fresh-cut CWC. This study also identified eriodictyol, naringenin, and sucrose as important features in this process, verifying the results of our previous study [21]. This investigation is a good starting point for providing a better theoretical understanding of how to efficiently inhibit the yellowing of CWC. However, further studies are needed for a deeper understanding of the metabolic pathways and enzymes that are associated with CWC yellowing.

## Figures and Tables

**Table 1:** The main identification obtained between 0 day and 2 days in ESI+ (*p* < 0.05).

VIP	RT (min)	Mass	Name	*T*-Test	Fold (2/0) *
1.248	13.14	278.2252	Linolenic Acid	0.001	+6.567
1.408	4.91	194.0582	ferulic acid	0.000	+4.237
1.779	4.06	297.09	5′-Deoxy-5′-(methylthio)adenosine	0.001	+4.040
1.479	6.60	548.1175	Chrysoeriol 7-*O*-(6″-malonyl-glucoside)	0.000	+2.683
1.204	10.19	357.2886	*N*-palmitoyl threonine	0.009	−2.457
1.843	8.06	276.2095	Stearidonic Acid	0.006	−2.086
1.137	4.33	137.0477	Anthranilic acid	0.000	−1.716
1.181	4.39	264.137	Abscisic Acid	0.038	−1.408
1.668	0.82	115.064	Proline	0.007	+0.275

* 0 day and 2 days is the logarithm of the ratio of the value of value (base 2), “+” means 2 days increase relative to 0 day, “−” means decrease. VIP: Variable Importance in the Projection; RT: retention time.

**Table 2:** The main identification obtained between 0 day and 2 days in ESI− (*p* < 0.05).

VIP	RT (min)	Mass	Name	*T*-Test	Fold (2/0) *
1.612	7.57	272.0694	Naringenin	0.001	+28.844
1.778	5.82	288.0642	Eriodictyol	0.000	+26.056
1.773	6.11	462.1179	Luteolin 5-methyl ether 7-glucoside	0.000	+24.638
1.325	11.24	479.3027	PE (18:1)	0.017	−24.315
1.759	13.13	278.2251	Linolenic Acid	0.000	24.309
1.153	3.47	712.1513	Quercetin 3-*O*-(6″-malonyl-glucoside) 7-*O*-glucoside	0.046	−24.296
1.624	4.87	304.0593	Pentahydroxyflavanone	0.001	+24.082
1.459	4.97	450.1182	Eriodictyol 5-*O*-glucoside	0.006	+23.416
1.398	10.03	475.271	PE (18:3)	0.010	−23.115
1.482	6.57	446.0862	Apigenin-glucuronide	0.005	+22.622
1.459	10.68	477.2872	PE (18:2)	0.006	−5.758
1.478	1.31	180.064	Glucose	0.005	−3.408
1.698	5.34	448.1027	Naringenin-4′-*O*-β-d-Glucuronide	0.000	+3.061
1.339	5.21	670.1768	Limocitrin 3,7-diglucoside	0.015	+2.829
1.480	4.48	176.0687	2-Isopropylmaleate	0.005	+2.556
1.245	1.11	668.1634	Euphorbianin	0.027	+2.481
1.634	4.91	180.0438	Caffeic Acid	0.001	+2.355
1.235	5.79	490.1128	Cyanidin 3-(6-acetylgalactoside)	0.029	+2.216
1.716	4.58	192.0629	Quinic acid	0.000	+1.921
1.515	5.58	207.0886	*N*-Acetyl-l-phenylalanine	0.003	−1.786
1.587	4.95	354.0962	Chlorogenic Acid	0.002	1.717
1.608	8.12	492.1279	Malvidin 3-*O*-glucoside	0.001	−0.310
1.369	5.69	168.042	Vanillic acid	0.012	−0.247
1.369	6.30	272.0687	Naringeninchalcone	0.012	−0.247
1.369	9.73	356.1271	Laurifolin	0.012	−0.247
1.369	5.78	534.1009	Cyanidin 3-*O*-(6-*O*-malonyl-β-d-glucoside)	0.012	−0.247
1.369	5.23	535.1341	Malvidin 3-(6-acetylglucoside)	0.012	−0.247
1.369	6.31	558.1173	Dihydromorelloflavone	0.012	−0.247
1.369	6.97	754.1943	Luteolin 7-glucuronide-3′,4′-dirhamnoside	0.012	−0.247
1.369	8.24	784.1795	Apigenin 7-Glucosyl-(1->2)-glucuronide-4′-glucuronide	0.012	−0.247

* 0 day and 2 days is the logarithm of the ratio of the value of value (base 2), “+” means 2 days increase relative to 0 day, “−” means decrease. VIP: Variable Importance in the Projection; RT: retention time.

**Table 3:** The main identification obtained between 2 days and 3 days in ESI+ (*p* < 0.05).

VIP	RT (min)	Mass	Name	*T*-Test	Fold (3/2) *
1.482	10.60	354.2777	MG (18:2)	0.023	−3.108
1.333	6.11	534.1018	Cyanidin 3-*O*-(6-*O*-malonyl-β-d-glucoside)	0.010	+2.596
1.081	1.04	174.0166	Aconitic acid	0.007	−2.281
1.037	5.95	374.0975	Dihydroxy-6,7,4′,5′-tetramethoxyflavone	0.022	+2.078
1.656	6.34	316.0954	Dihydroxy-dimethoxyflavanone	0.018	+0.879
1.449	0.82	115.064	Proline	0.019	−0.282

* 2 days and 3 days is the logarithm of the ratio of the value of value (base 2), “+” means 3 days increases relative to 2 days, “−” means decrease. VIP: Variable Importance in the Projection; RT: retention time.

**Table 4:** The main identification obtained between 2 days and 3 days in ESI− (*p* < 0.05).

VIP	RT (min)	Mass	Name	*T*-Test	Fold (3/2) *
1.289	4.578	192.0634	Quinic acid	0.019	+25.997
1.558	3.970	191.0619	*N*-Acetyl-dl-methionine	0.002	+25.741
1.810	6.100	448.1022	Naringenin-4′-*O*-β-d-Glucuronide	0.000	+24.727
1.830	5.969	332.0543	Quercetagetin-methyl ether	0.000	+24.325
1.295	9.730	356.1271	Laurifolin	0.018	+23.249
1.739	5.783	534.1009	Luteolin 5-(6″-malonylglucoside)	0.000	+22.968
1.588	5.234	535.1341	Malvidin 3-(6-acetylglucoside)	0.001	+22.258
1.264	5.144	358.068	Dihydroxy-dimethoxy-methylenedioxy	0.022	+21.350
1.200	7.953	610.0885	Gallocatechin 7,4′-di-*O*-gallate	0.032	+21.183
1.361	10.026	475.271	PE (18:3)	0.011	+21.183
1.779	5.245	318.0388	Myricetin	0.000	+6.475
1.254	11.404	532.1541	Flavonol 3-*O*-d-xylosylglucoside	0.023	+5.586
1.422	5.464	534.0963	Cyanidin 3-*O*-(6-*O*-malonyl-β-d-glucoside)	0.007	+4.072
1.737	5.877	218.1155	Pantothenic Acid	0.000	+3.618
1.757	7.131	302.0438	Quercetin	0.000	+3.545
1.323	8.530	286.0838	Naringenin 5-methyl ether	0.015	+3.037
1.606	4.247	304.0591	Pentahydroxyflavanone	0.001	+2.656
1.342	6.523	476.1319	Naringenin 7-*O*-beta-d-glucoside 6″-acetate	0.013	+2.508
1.399	5.708	450.1176	Eriodictyol-*O*-glucoside	0.008	+2.388
1.577	1.056	176.0325	Ascorbic acid	0.001	−2.228
1.395	13.135	278.2251	Linolenic Acid	0.009	+1.268
1.123	5.787	490.1128	Cyanidin 3-(6-acetylgalactoside)	0.049	+1.194
1.338	4.912	180.0438	Caffeic Acid	0.013	−1.091
1.312	0.721	422.0835	Sucrose-6-phosphate	0.016	−0.608

* 2 days and 3 days is the logarithm of the ratio of the value of value (base 2), “+” means 3 days increases relative to 2 days, “−” means decrease. VIP: Variable Importance in the Projection; RT: retention time.

**Table 5:** The main identification obtained between 3 days and 4 days in ESI+ (*p* < 0.05).

VIP	RT (min)	Mass	Name	*T*-Test	Fold (4/3) *
1.383	7.64	300.064	Trihydroxy-3-methoxyflavone	0.010	−3.618
1.039	6.34	316.0954	Dihydroxy-7,2′-dimethoxyflavanone	0.000	+1.796
1.155	10.43	354.2779	MG (18:2)	0.037	−1.697
1.899	11.12	356.2934	MG (18:1)	0.036	−1.169
1.333	4.02	117.0579	Indole	0.010	+0.508
2.041	10.39	198.1623	Lauroleic acid	0.029	+0.304

* 3 days and 4 days is the logarithm of the ratio of the value of value (base 2), “+” means 4 days increases relative to 3 days, “−” means decrease. VIP: Variable Importance in the Projection; RT: retention time.

**Table 6:** The main identification obtained between 3 days and 4 days in ESI− (*p* < 0.05).

VIP	RT (min)	Mass	Name	*T*-Test	Fold (4/3) *
2.120	8.67	588.1278	Kolaflavanone	0.000	+5.933
2.104	9.73	356.1271	Laurifolin	0.000	+3.667
1.516	1.09	433.1176	Cyanidin 3-rhamnoside	0.034	+3.486
1.579	1.32	216.040	2-*C*-Methyl-d-erythritol 4-phosphate	0.026	−3.477
2.215	4.87	138.0319	Salicylic acid	0.000	+2.569
1.871	5.50	302.0797	Ferreirin	0.004	+2.537
1.518	0.68	250.153	Xanthoxin	0.034	−2.407
2.173	7.52	316.0956	Eriodictyol 7,3′-dimethyl ether	0.000	+2.239
2.241	5.69	168.042	Vanillic acid	0.000	+2.192
1.664	7.95	610.0885	Epigallocatechin 5,7-di-*O*-gallate	0.017	+2.162
2.028	5.14	358.068	Dihydroxy-dimethoxy-methylenedioxy	0.001	+1.843
2.096	3.97	191.0619	*N*-Acetyl-dl-methionine	0.001	+1.830
1.554	4.97	450.1182	Eriodictyol 6-*O*-glucoside	0.029	+1.777
1.878	5.48	448.1021	Naringenin 5-*O*-glucuronide	0.004	+1.723
1.765	7.94	316.0593	Quercetin 3-methyl ether	0.009	+1.717
1.565	5.82	288.0642	Eriodictyol	0.027	+1.613
1.683	8.53	286.0838	Naringenin 5-methyl ether	0.015	+1.450
1.621	6.60	136.0522	Phenylacetic acid	0.021	−1.387
2.032	5.25	318.0388	Myricetin	0.001	+1.256
1.784	4.58	192.0634	Quinic acid	0.008	+1.190
1.640	13.13	278.2251	Linolenic Acid	0.019	−1.021

* 3 days and 4 days is the logarithm of the ratio of the value of value (base 2), “+” means 4 days increases relative to 3 days, “−” means decrease. VIP: Variable Importance in the Projection; RT: retention time.

**Table 7:** The main identification obtained between 4 days and 5 days in ESI+ (*p* < 0.05).

VIP	RT (min)	Mass	Name	*T*-Test	Fold (5/4) *
1.222	11.44	315.278	Dehydrophytosphingosine	0.023	+22.358
1.547	13.14	278.2252	Linolenic Acid	0.000	+2.291
1.106	10.43	354.2779	MG(18:2)	0.017	+1.874
1.337	6.98	708.189	Patuletin 3,7-(3-acetylrhamnoside)	0.004	+1.359
1.110	7.64	300.064	Trihydroxy-3-methoxyflavone	0.032	+1.167
1.317	0.66	155.0697	Histidine	0.003	-0.467

* 4 days and 5 days is the logarithm of the ratio of the value of value (base 2), “+” means 5 days increases relative to 4 days, “−” means decrease. VIP: Variable Importance in the Projection; RT: retention time.

**Table 8:** The main identification obtained between 4 days and 5 days in ESI− (*p* < 0.05).

VIP	RT (min)	Mass	Name	*T*-Test	Fold (5/4) *
1.442	6.18	448.1026	Naringenin-4′-*O*-β-d-Glucuronide	0.015	−23.434
1.259	1.06	176.0325	Ascorbic acid	0.042	−3.930
1.828	4.39	130.063	Ketoleucine	0.000	+2.189
1.840	13.13	278.2251	Linolenic Acid	0.000	+2.116
1.640	7.07	264.1366	Abscisic Acid	0.003	+2.097
1.954	5.23	535.1341	Malvidin 3-(6-acetylglucoside)	0.000	+2.016
1.503	5.21	670.1768	Limocitrin 3,7-diglucoside	0.010	+1.913
1.515	4.78	122.0368	Salicylaldehyde	0.009	−1.547
1.436	13.73	280.2405	Linoleic acid	0.016	1.395
1.694	1.34	131.0952	Leucine	0.002	−1.374
1.580	7.90	302.0799	Ferreirin	0.005	+1.275
1.238	3.96	354.0963	Chlorogenic Acid	0.046	−1.241
1.228	6.74	270.0534	Trihydroxyflavone	0.048	−1.213
1.843	5.69	168.042	Vanillic acid	0.000	+1.072
1.807	0.69	155.0699	Histidine	0.000	−0.979
1.951	2.22	148.0526	Cinnamic acid	0.000	−0.827
1.280	9.73	356.1271	Laurifolin	0.038	+0.821
1.476	4.58	376.0789	Tetrahydroxy-3,6,2′-trimethoxyflavone	0.012	−0.815
1.259	8.67	588.1278	Kolaflavanone	0.042	+0.763
1.352	5.89	194.0581	ferulic acid	0.026	−0.639
1.543	4.01	117.0581	Indole	0.007	−0.609
1.493	0.74	147.0545	Glutamate	0.011	−0.503
1.610	4.58	422.0842	Sucrose-6-phosphate	0.004	−0.513

* 4 days and 5 days is the logarithm of the ratio of the value of value (base 2), “+” means 5 days increases relative to 4 days, “−” means decrease. VIP: Variable Importance in the Projection; RT: retention time.
